# CEMTDD: The database for elucidating the relationships among herbs, compounds, targets and related diseases for Chinese ethnic minority traditional drugs

**DOI:** 10.18632/oncotarget.3789

**Published:** 2015-04-20

**Authors:** Jian Huang, Yaxin Zheng, Wenxi Wu, Tao Xie, Hong Yao, Xiaobo Pang, Fuzhou Sun, Liang Ouyang, Jinhui Wang

**Affiliations:** ^1^ Key Laboratory of Structure-Based Drug Design & Discovery of Ministry of Education, School of Traditional Chinese Materia Medica, Shenyang Pharmaceutical University, Shenyang, China; ^2^ State Key Laboratory of Biotherapy, Collaborative Innovation Center of Biotherapy, West China Hospital, Sichuan University, Chengdu, China; ^3^ School of Pharmacy, Shihezi University, Shihezi, China

**Keywords:** chinese ethnic minority traditional drug (CEMTD), xinjiang uygur autonomous region, database, herb, gerontology

## Abstract

China has different ethnic minorities that establish their own medical systems and practice experience for thousand years, thereafter named Chinese Ethnic Minority Traditional Drugs (CEMTDs) (http://www.cemtdd.com/index.html). Since many compounds from CEMTDs have been reported to perturb human's dysfunction network and restore human normal physiological conditions, the relationships amongst a series of compounds from specific herbs, their targets and relevant diseases have become our main focus in CEMTD modernization. Herein, we have constructed the first Chinese Ethnic Minority Traditional Drug Database (CEMTDD) mainly from Xinjiang Uygur Autonomous Region (XUAR), retrieving CEMTD-related information from different resources. CEMTDD contains about 621 herbs, 4, 060 compounds, 2, 163 targets and 210 diseases, among which most of herbs can be applied into gerontology therapy including inflammation, cardiovascular disease and neurodegenerative disease. Gerontology is highly occurred in XUAR, and has abundant experience in treating such diseases, which may benefit for developing a new gerontology therapeutic strategy. CEMTDD displays networks for intricate relationships between CEMTDs and treated diseases, as well as the interrelations between active compounds and action targets, which may shed new light on the combination therapy of CEMTDs and further understanding of their herb molecular mechanisms for better modernized utilizations of CEMTDs, especially in gerontology.

## INTRODUCTION

Chinese Ethnic Minority Traditional Drugs (CEMTDs), originated from different nations in China, have been widely used as therapeutic regimen for thousands of years [1]. Currently, CEMTDs have been playing their important roles in maintaining health of Chinese minority people, and drawing much more attention around the world. These CEMTDs have been widely used to treat almost all human diseases, especially gerontology diseases, such as cancer, inflammation, cardiovascular disease and neurodegenerative diseases etc. However, CEMTDs are basically based upon some unpopular theories in the minority area, which are different from the philosophy of both modern western medicine and traditional Chinese medicine (TCM); thereby, they are largely prevented from being recognized by the mainland of China. To facilitate the research on CEMTDs, it is essential to combine ancient practices of CEMTDs with modern standards, especially focusing on exploring herb molecular mechanisms in their treatment [2].

In the past few decades, some Chinese scientists have made their great efforts to explore various CEMTDs and decompose their isolated bioactive compounds, in which different action targets are also identified. These findings may provide precious resources and useful guidance for developing and utilizing CEMTDs to treat human diseases at the system level. Besides, CEMTDs can improve the efficacy of treatment from synergistic interactions amongst a various number of compounds. Moreover, we may extract different kinds of compounds from herbs, and these compounds have their own targets; thus this relation relates herbs to diseases. Thus, the links between CEMTDs and their targets in different diseases may provide useful information to demystify the theory underlying treatment of CEMTDs [3, 4]. To truly modernize CEMTDs, current systematic methods should be updated, and active compounds in CEMTDs should all be simultaneously taken into consideration [5]. Additionally, current strategy for drug discovery is mainly focusing on the “one gene-one drug-one disease” code, which has caused limitations since the drug's efficacy is impaired by the robustness of protein-protein interaction (PPI) network in the treated objectives [6]. To overcome such limitations, there is an urgent need to use systems-oriented approaches for effective combinatorial drugs. Therefore, turning to CEMTDs may be a sensible solution because they may treat diverse diseases in a holistic way.

Considering the above-mentioned reasons, we built this CEMTD-integration database, especially focusing on Kazakhstan and Uygur drugs, with big data from different aspects, such as herbs, compounds, targets and diseases [7]. Moreover, since both TCM and CEMTDs can be utilized to treat diseases by applying compounds to interact with disease-specific function networks to maintain human health, this common aspect can be used as a fundamental factor to bridge the gap between these two groups of traditional drugs in China [8, 9].

As a general term to describe diseases that are specific or common diseases in the elders, gerontology is associated to features of aging and diversified across different locations, environment and climate [10]. With the increase of life expectancy and the steadily growing of aging population worldwide, the study of aging and gerontology therapy have drawn extensive attention for the recent years [11]. The most common gerontology includes Cardiovascular Disease, Inflammations, Neurodegenerative Disease and Cancer etc., which have been researched and implemented for thousands of years by practicing CEMTDs. Utilizations of CEMTDs in geriatric treatment with minor side effects are worthy of further investigation for future development of gerontology therapy, however, systematic elucidation and specific target of CEMTDs in gerontology remain to be uncovered [12].

In this study, we constructed CEMTDD for the first time to collect a wealthy number of data about CEMTDs from' Xinjiang Uygur autonomous region. And through comprehensive integration of various data and information, our CEMTDD records a wealthy of information especially from Kazakhstan and Uygur drugs, in different aspects including herbs, compounds, targets, and diseases, indicating how herbal compounds regulate their specific targets that are related to different diseases (herb molecular mechanisms); thereby this database may provide more new clues on future combination therapy and further better utilizations of CEMTDs, especially in gerontology therapy.

## RESULTS

### Database query

**Figure 1:**
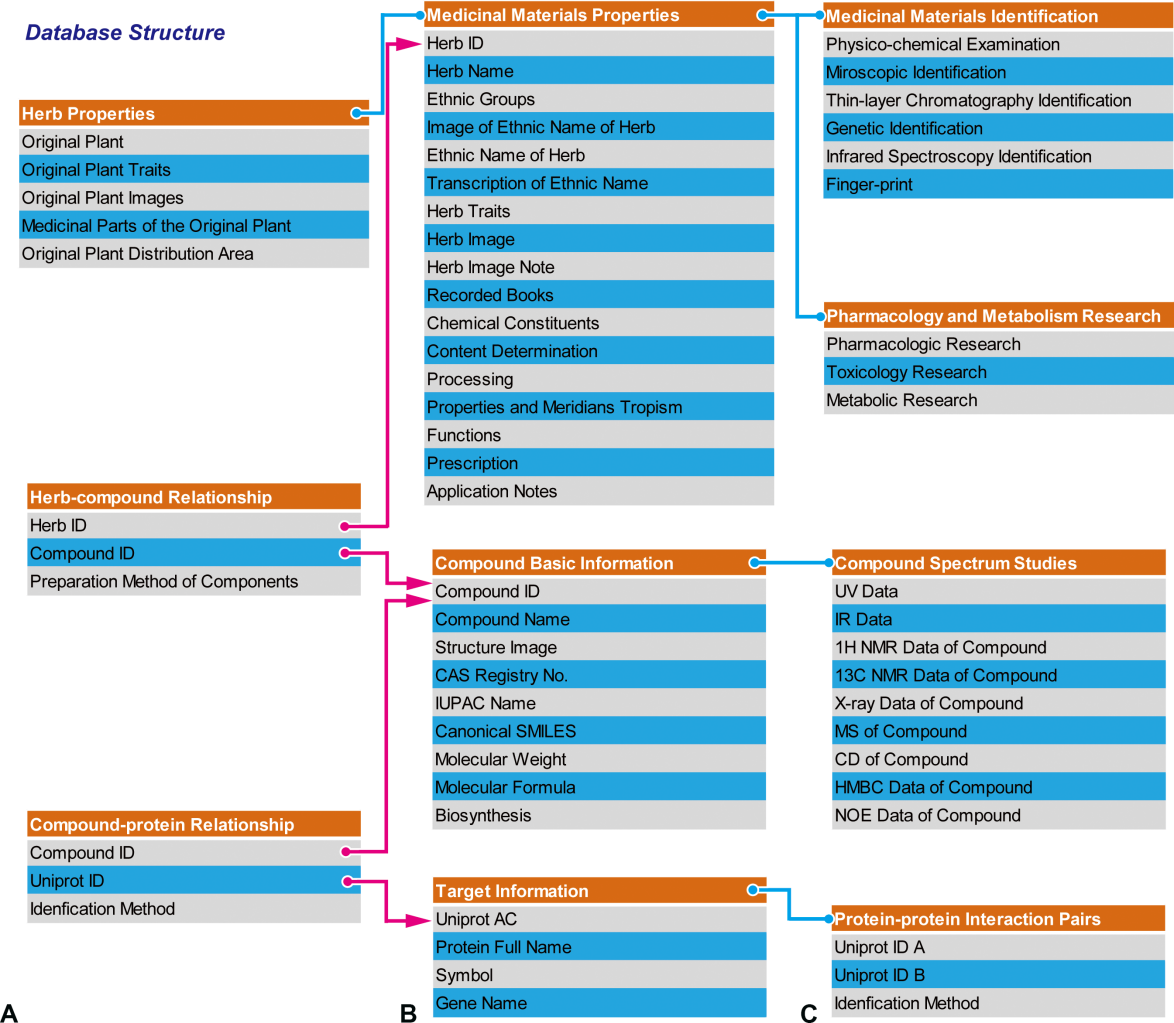
Database structure **A.** The structure of the database is mainly composed of herb properties, herb-compound relationship, and compound-protein relationship. **B, C.** All of these features could be further trivialized and interconnected with each other.

CEMTDD has collected all information resources from four different intricately connected fields, such as herbs, compounds, targets and diseases. Users can use any data field to query the database and follow the link to retrieve related information. For example, users can enter the serial number or the herb name to query information of a certain compound. The result page will not only show the related information of the inquired targets, but corresponding information about herbs, compounds, targets and treated diseases. Additionally, users can use these provided hyperlinks to search for further detailed information. Besides, the hyperlinks of compounds, targets and diseases can also be linked to other databases, such as UniProt, OMIM and *etc*. (Figure 1A).

### Network display

To virtually screen the relationships among herbs, compounds, targets and diseases, we have developed network-display tools, which provide more detailed network information. We also constructed PPI network to help users to check the potential combinational effects. After integrating data PPI network for targets, users can get a visually global connection blueprint of the “herb-compound-target-disease” standard (Figure 1B).

### Herb-disease network

In traditional medical science of Chinese minority, different types of herbs have been widely used to treat several specific diseases for a long time, even though the exact mechanisms are not clearly elaborated. Thus, it is beneficial to link herbs and their treated diseases. We established the schema of herbs and their corresponding traditional medicine theories; thereby drawing a blueprint of “herb-disease” relation (Figure 1C).

**Figure 2:**
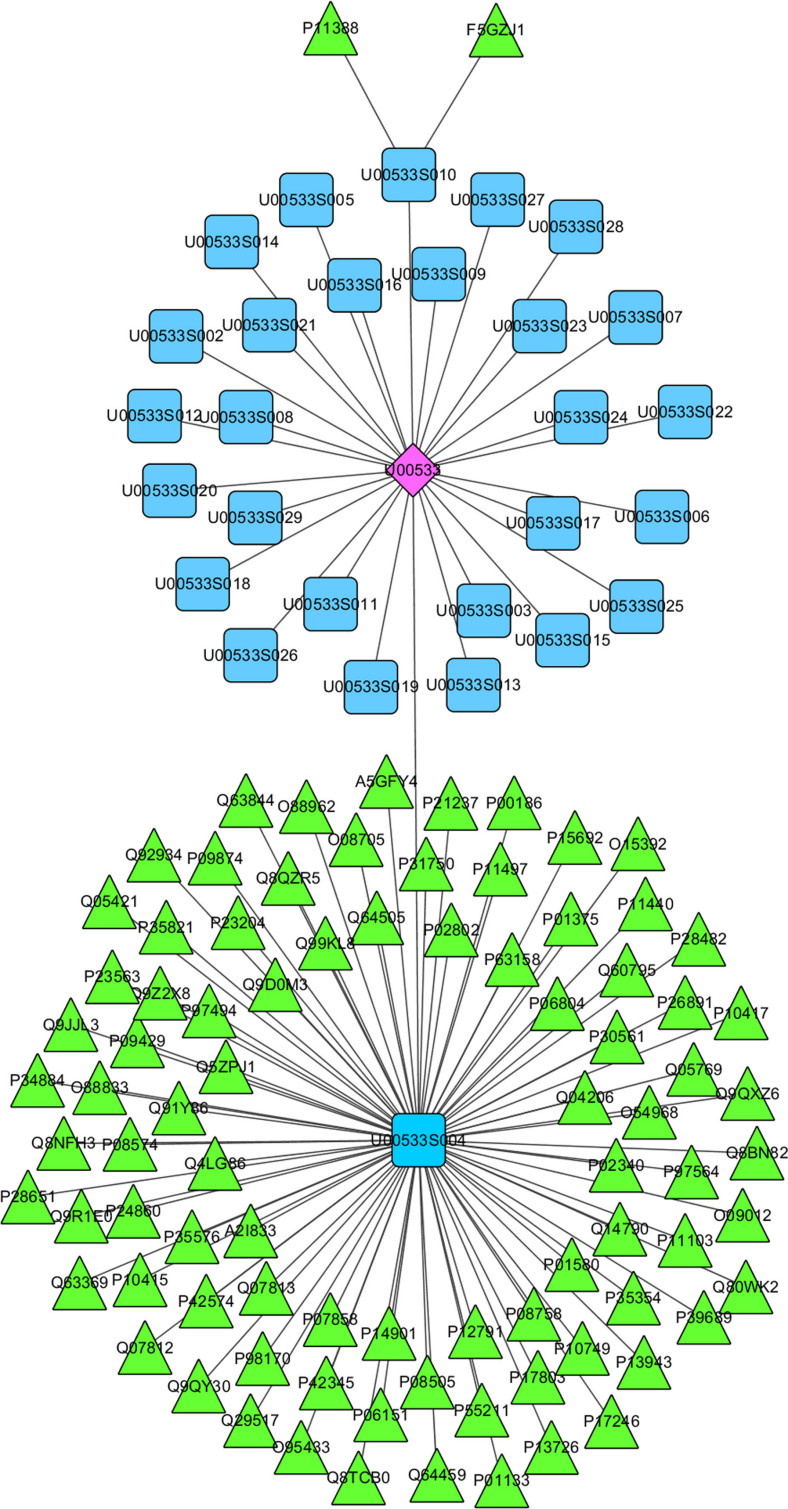
The Kazakh drug Salvia deserta Schang serves as an example for the use of CEMTDD web interface to find its target information The CEMTDD web interface provides target information of different herbs, taking *Salvia deserta* Schang as an example, U00533 is the recorded number of *Salvia deserta* Schang in CEMTDD, U00533S001-U00533S029 represent compounds of *Salvia deserta* Schang. Protein targets of each compound are graphical represented by AC number.

**Figure 3:**
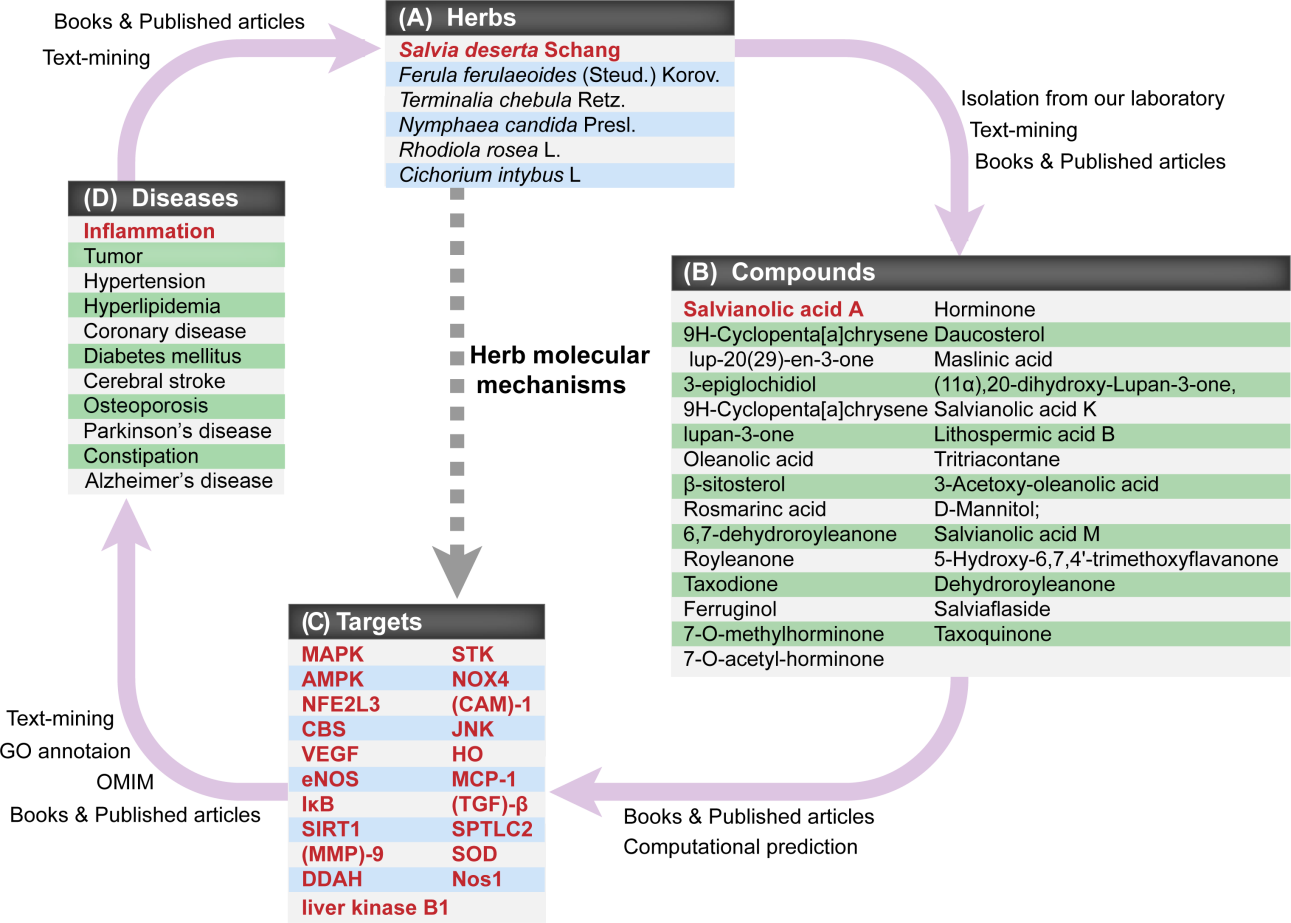
The interrelationships among CEMTDs, chemical compounds, targets and treated diseases Relationships of four key modules in CEMTDD are described.

For instance, our database has collected studies on the identification of medical materials in *Salvia deserta* Schang, including traditional functional treatment, prescription application, pharmacology and toxicity (Figure 2). As previously reported, *S*. *deserta* Schang can be used in the treatment of cardiovascular and cerebrovascular diseases, cancer, diabetes, inflammation and *etc*. Meanwhile, we offered the physicochemical property and spectrum data of 28 compounds contained in *S*. *deserta* Schang, mainly including 9 triterpenes, 9 diterpenes, and 5 phenolic acids. Twelve of these 28 compounds, such as Oleanolic acid, Ursolic acid, β-sitosterol, Rosmarinc acid, Salvianolic acid A, Taxodione, Ferruginol, Daucosterol, Maslinic acid, Lithospermic acid B, and D-Mannitol, have been reported to exert biological functions that are closely linked to inflammation, cell cycle, apoptosis, and oxidative stress. Salvianolic acid A (SAA) and Lithospermic acid B are key two that have multiple targets. For example, SAA can target mitogen-activated protein kinase (MAPK), AMP-activated protein kinase (AMPK), Nuclear factor erythroid 2-related factor 3 (NFE2L3), cystathionine β-synthase (CBS), cystathionase, vascular endothelial growth factor (VEGF), gelatinase A, nitric oxide synthase (NOS), inhibitor of NF-κB (IκB), sirtuin-1 (SIRT1), caspase-3, matrix metalloproteinase (MMP)-9, dimethylarginine dimethylaminohydrolase (DDAH), liver kinase B1, serine-threonine protein kinase (STK), NADPH oxidase 4 (NOX4), Bcl-2, and cell adhesion molecule (CAM)-1 [23–27]. Lithospermic acid B aims at some protein targets such as c-Jun N-terminal kinases (JNKs), caspase-3, heme-oxygenase (HO), SIRT-1, monocyte chemoattractant protein-1 (MCP-1), transforming growth factor (TGF)-β [28, 29, 30, 31]. Moreover, other compounds including Taxodione and Ferruginol can target Bax, caspase-3 and PARP1 or caspase-8, AIF and PI3K, respectively [32, 33] (Figure 3). These above-mentioned data have revealed that an herb, like *S. deserta Schang*, may contain a number of compounds, which have similar or specific targets in above-mentioned human diseases.

### Gerontology therapeutic implications in CEMTDD

In this database, we have integrated PPI data from several protein interaction databases to this network; thereby, users may find whether different targets in different herbs can be inner-connected. In addition, with integration of data about genes and diseases from different databases, we have connected herbs to diseases under the guidance of the “herb-compound-target-disease” standard. Users may use these potential connections to find possible novel therapeutic effects of different herbs, or new herbs for the same disease. Herein, the expected types of Kazakhstan and Uygur traditional drugs are over 600; however, there are 152 types of true medical materials, and the drugs that we have extracted compounds from are up to 370 types. In addition, the number of compounds reaches 4, 060 with an average of 10.9 in each medicinal material. Through searching data from text mining and several online databases, we have found 491 compounds with specific targets, which account for 12.1% in all compounds (Figure 1).

Gerontology is considerably related to the process of aging, which participates in either the promotion of degenerative pathologies that correspond to the losses biological functions, or the promotion of hyperplastic pathologies such as cancer. Including nutrient sensors such as the kinase mTOR, AMPK, SIRT and IGF-1, stress response genes like HSF-1 and HIF-1, different signaling pathways have been revealed involving in the process of aging and thus may cause gerontology [34]. Concerning the increasingly complicated interaction of proteins in gerontology, a convergence of methods in both biomedical science and database retrieving are essential for gerontology therapy development. From herbs to diseases, CEMTDD may bridge the gap between national herbs and gerontology, especially in some well-studied common gerontology diseases, such as neurodegenerative diseases, cardiovascular disease and cancers.

One of the most consistent risk factor for developing neurodegenerative diseases is aging, which often comes a decline volume and function of brain or permanent loss of neurons, attributing to the neurodegenerative disease, such as Alzheimer's Diseases, Parkinson's disease and amyotrophic lateral sclerosis [35]. For instance, *Ferula ferulaeoides*, record number in CEMTDD is U00136, was found interacted with neprilysin, currently conceived as major enzyme for degradation of Aβ in Alzheimer's Diseases [36]. Extract of *Rhodiola rosea L*. *(U00648)*, Salidroside, is reported to exert protective effects of in the MPTP/MPP(+)-induced model of Parkinson's disease through ROS-NO-Related mitochondrion pathway [37].

Meanwhile, the incidence of cardiovascular disease (CVD), such as schemic heart disease (IHD), stroke and hypertensive heart disease, increases with age [38]. In CVD, regulation of blood pressure, regulation of vasculogenesis and nitric oxide biosynthetic process are selected for targets screening, while many proteins associated with CEMTDs were found. *Cichorium intybus L*., U00249, used to be applied for its implication of hepatic disorders and dyspepsia therapy in CEMTD; however, its compound U00249S053 is in correlation with Prostaglandin G/H synthase 2, annotated for regulation of blood pressure, and may have CVD therapeutic applications [39]. Moreover, a well-known herbs in treating inflammation, such as pharyngitis and pneumonia, *Terminalia chebula Retz* (U00184) is found associated with Sepiapterin reductase, an enzyme that responsible for nitric oxide biosynthetic process, thus, it may also be applied in CVD treatment [40].

Furthermore, CEMTDD could provide us with clues in cancer treatment. Several cancer associated proteins, such as p53, caspase and Chk2, all map well in one or several compounds within these herbs such as *Rhodiola rosea L.(U00648)*, *Cichorium intybus L. (U00249)* and *Ferula ferulaeoides (U00136)*, among which *Rhodiola rosea L*. is widely used in traditional medicine in Uyghur, renowned for its efficacy in work performance improvement and fatigue remission. Now researches in lung cancer A549 cells and fibrosarcoma HT1080 cells revealed the anti-tumor capacity of *Rhodiola rosea L*. compound Salidroside [41, 42].

Due to the systematic complexity and a progressive decline of many physiological functions of the elders, diseases of gerontology are normally synergistic and face clinical treatment difficulties. Therefore, therapy of gerontology requires multi-target and combination therapy, which is corresponded to CEMTD strategy: “Multi-components, Multi-target and Collaborative treatment”. Most of herbs in CEMTDD have been reported or practiced to treat common diseases of gerontology, and based on the integrated information of herbs, compounds, targets, diseases and PPI network in CEMTDD, our study may provide with in-depth clues regarding how the compounds are interacted with established bio-gerontology processes through the presumed targets.

## DISCUSSION

Hitherto, different nations from China have had their own biomedical theories and systems, and the empirical evidence-based theories and systems have contributed a lot to the health of Chinese minority people; thereby, medical resource from nature is our huge treasure. And a thoroughly modernized database of most ethnic traditional drugs is necessary for elucidating CEMTDs systematically; accordingly, CEMTDD contains plants (medicinal materials), metabolites, indications, compounds, target proteins, molecular mechanisms and other related information of CEMTDs, integrating to establish a serviceable source of natural products to benefit drug discovery.

Uygur traditional drugs, an important component of Islamic Medicine, are normally used in Xinjiang Autonomous Region Range. According to Uygur medicine Blog, there are 124 kinds from more than 600 Uygur traditional drugs that are usually used by Uygur people. Additionally, Kazakh Drug Blog has established a comparatively scientific and intact theory system, suggesting that origins, proliferation, development and death of human beings have their own specific substances. For long-time clinical practices, Uygur and Kazakh people have found many suitable drugs according to their different gene polymorphism and epigenetics especially in the fields such as blood fat, tumor, heart and cerebral diseases, as well as metabolic enzymes [43, 44]. Therefore, through systematical mining, we have provided a number of new targets and relevant drugs especially for Uygur and Kazakh traditional drugs. Since Uygur and Kazakh people have a faith in Mohammedanism, they usually use oil instead of wine to prepare drugs, among which the oil processed Rosa rugosa can be used for improving sleep. Due to their own cultural transmission and habitation of different nations, there are obvious distinctions in national medical components of different ethnic minority traditional drugs. For instance, Uygur traditional drugs are greatly influenced by the culture of Persian and Islamic, and thus most of these drugs are from central Asia and minor Asia, while Kazakh drugs, such as *Ferula ferulaeoides* (*Steud*.) *Korov* and *Rhodiola rosea L* are mainly based upon local resources of XUAR from Mount Tianshan, Mount Aertai and Junggar Basin. Although Uygur and Kazakhstan traditional drugs have a long history of pharmacy and have been used in clinical practices to help many patients, however, their underlying mechanisms and pharmaceutical effects were still poorly understood. Thus, developing modernized medicinal methods and tools to explain drug action foundation of CEMTDs is urgently needed.

To develop these ethnic traditional drugs and improve public recognition about them, CEMTDD aims to systematically collect research of different aspects, including sources, chemical compounds, therapeutic targets, and treatment of diseases. Besides, we have collected physicochemical properties, biological activities and active targets mainly by text mining from books, published articles and online databases; thereby, the relations between chemical compounds of specific drugs and their disease-related protein modulations are established. We have also applied different computational methods, such as SEA DOCK, Reversing Docking, Cytoscape Web and PrePPI to predict select related compounds and potential targets. Therefore, we have established the standard of “herb-compound-target-disease” to construct our CEMTDD. Moreover, our database has collected the drug use of ethnic minority traditional drugs under the guidance of Uygur drug and Kazakh drug theories.

As far as we concern, our database will provide ethnic traditional drug researchers with systematical search and “herb-compound-target-disease” standard; thereby exerting an influence on different parts. Firstly, CEMTDD may provide a reference to clinical medication of ethnic drugs with system information of chemical compounds and pharmacology. Herbs with the same compounds or targets may have inner connections and similar functions, accordingly, the relation between those herbs and offering a theoretical foundation of combination therapy. For example, the whole plant of Kazakh drug *Salvia deserta Schang* can clear heating and toxic materials, exert anti-bacterial and anti-inflammatory effects, resist diabetes, reduce fever, relieve pain, and reduce phlegm. Some papers have reported that *S. deserta Schang* and *Salvia miltiorrhiza Bge* hold similar effects in the same diseases such as cardiovascular and cerebrovascular diseases, since both of them can be used for promoting blood circulation and removing blood stasis and pain. So, it is reasonable to believe *Salvia deserta Schang* may replace *S. miltiorrhiza* to treat CVD by targeting same genes/proteins. Many different targets have been studied thoroughly in *S. miltiorrhiza*, and such targets are also found in *S. deserta Schang*. We have collected information about these targets in CEMTDD, thereby providing an important foundation for clinical doctors to take advantage of *S. deserta Schang*, which shares similar features with *S. miltiorrhiza Bge*. Additionally, CEMTDD can provide fundamental data in different aspects, such as ethnic traditional drug theory, modernized medication system, and effective element information with their direct or indirect targets for pharmacologists. Thirdly, CEMTDD can offer specific targets in different drugs and their related functions. CEMTDD has also developed robust function in finding clues for specific disease in aid of GO annotation. To facilitate the study of gerontology, several targets were confirmed associated with specific chemicals within four different herbs. The study of gerontology may largely include cancer, neurodegenerative diseases, and cardiovascular diseases.

Generally, CEMTDD provides us a basis for a bridge between western medicine and ethnic medication by drug screening with bioinformatics approaches. It also promotes the development of ethnic traditional drugs while popularizes their information about research progresses and medication of ethnic traditional drugs. In this database, 112 from 621 ethnic minority traditional drugs have also be recognized and utilized as traditional Chinese medicines (TCMs). Compared to TCM, some ethnic minority traditional drugs absorb the essence of TCMs, but most drugs are exclusive in their own nations (509 from 621 ethnic minority traditional drugs), with their own national features of application. For example, *Veratrum nigrum* is mainly used for promoting emesis in TCMs, while as a Uygur traditional drug it can be used for treating hearing loss. Moreover, the nomadic life of Kazakh people has made them vulnerable to rheumatic arthritis, fall damage, malnutrition, and altitude stress; thereby their ethnic traditional drugs have more prominent characteristics in the related fields. Different epigenetics may cause special characteristics, and thus CEMTDs may be beneficial for future personalized therapeutics. Therefore, this database would provide a new insight into the herb molecular mechanisms for better CEMTDs modernization from Xinjiang Uygur autonomous region.

## MATERIALS AND METHODS

### Data collection

CEMTDD, especially focusing on Kazakhstan and Uygur traditional drugs, is comprised of four data fields, including herbs, compounds, targets and diseases. The information and data in these different fields were integrated from related web-based databases and text mining of books and published articles.

The information of herbs, such as name and structure, was collected mainly through text-mining methods from books and published articles including Kazakh drug Blog, Uygur medicine Blog, Chinese Pharmacopoeia and Uygur medicine branch released by ministry of health in P.R. China. For the data field of compounds, we collected most corresponding information from a series of online databases, such as CNKI, SCI finder, web of science and Google, including published papers in both English and Chinese. Importantly, our lab has isolated, purified, and identified a series of bioactive compounds, for which we have proved structures and action mechanisms, thus providing a supplement to the current known compounds retrieved from online databases. We have also used Similarity Ensemble Approach (SEA) DOCK [13] to predict a series of potential targets besides from the already existed targets. In addition, we converted the information of targets into UniProt accession number, which is a comprehensive, high-quality and freely accessible resource of protein sequence and functional information (Table 1).

**Table 1:** Data resource

Data field	Data source	Amount of data
**Herb**	Kazakh medicine Blog, Uygur medicine Blog, Chinese Pharmacopoeia, and Chinese Materia Medica (Uygur medicine volume), text-mining	621
**Compound**	CNKI, SCI finder, Web of Science, Google, Text-mining	4, 060
**Target**	SEA Dock, Google, Text-mining	2, 163
**Disease**	PubMed, CNKI, SCI finder, Web of Science, Google, Text-mining	210

### Data integration

The goal of our database was to build connections between herbs (including herbal compounds) and diseases through disease-related genes/proteins (potential drug targets). Accordingly, we have applied different methods as follows:

Firstly, besides from using methods such as text mining and searching for data from online databases, we have predicted some potential targets of compounds from SEA DOCK, and Reversing Docking [14, 15]. The similarity score between each set was calculated by ligand topology. By inputting the compound's SMILES and a specific identifier, target names, reference names, scores and structures can be obtained. Moreover, we have developed an assistant tool to simplify this DOCK process, and get results more quickly and easily. Using Reversing docking, we ranked the potential targets of the compounds by Libdock score. The targets were downloaded from sc-PDB, an annotated database of druggable binding sites from the Protein DataBank [16].

Subsequently, the information from different books and published articles in Kazakhstan and Uygur traditional drugs and related results were mainly published in Chinese; thereby, we collected and manually extracted the related data of compounds and their targets. In total, we collected 4, 060 compounds, and 491 of them have specific targets from diverse sources. We also recorded descriptions for experimental evidence, related URL, and title for each article. The result of SEA Dock also shows that there are N computational predicted targets for these 491 compounds.

Then, we established the related protein-protein interaction (PPI) network for the data field of all the targets. We firstly extracted useful information of targets by text-mining methods from some online databases including Uniprot and OMIM [17, 18]. Subsequently, we collected diverse PPIs from DIP [19], IntAct [20], and PrePPI [21], and built PPI network for both known and predicted targets. We have also taken advantage of the network tool Cytoscape Web [22], and thereby generating visual network.

The four data fields in our database system are inter-connected with their intrinsic relations depending on the standard of “herb-compound-target-disease” (Figure 1): an herb is composed of compounds, a compound can interact with its target, and a disease may be caused by the dysfunction of genes/proteins.
